# Glycyrrhizic Acid Promotes Osteogenic Differentiation of Human Bone Marrow Stromal Cells by Activating the Wnt/β-Catenin Signaling Pathway

**DOI:** 10.3389/fphar.2021.607635

**Published:** 2021-04-16

**Authors:** Jinwu Bai, Jianxiang Xu, Kai Hang, Zhihui Kuang, Li Ying, Chenwei Zhou, Licheng Ni, Yibo Wang, Deting Xue

**Affiliations:** ^1^ Department of Orthopaedics, The Second Affiliated Hospital, Zhejiang University School of Medicine, Zhejiang University, Hangzhou, China; ^2^ Orthopedics Research Institute, Zhejiang University, Hangzhou, China

**Keywords:** glycyrrhizic acid, osteogenesis, Wnt/β-catenin, GelMA hydrogels, human bone marrow stromal cells

## Abstract

Glycyrrhizic acid (GA) is a major triterpene glycoside isolated from liquorice root that has been shown to inhibit osteoclastogenesis. However, there have been no reports regarding the effect of GA on osteogenic differentiation. Therefore, this study was performed to explore the effects and mechanism of action of GA on osteogenesis. A CCK-8 array was used to assess cell viability. The osteogenic capability was investigated by real-time quantitative PCR, western blotting and immunofluorescence analyses. ALP staining and ARS were used to evaluate ALP activity and mineralization, respectively. GA-GelMA hydrogels were designed to verify the therapeutic effects of GA *in vivo* by radiographic analysis and histological evaluation. Our results show that GA had no significant influence on the viability or proliferation of human bone marrow stromal cells (hBMSCs). GA promoted osteogenic differentiation and enhanced calcium deposition. Furthermore, ratio of active *β*-catenin and total *β*-catenin protein increased after treatment with GA. Wnt/catenin signaling inhibitor partially attenuated the effects of GA on osteogenic differentiation. In a mouse femoral fracture model, GA-GelMA hydrogels accelerated bone healing. Our results show that GA promotes the osteogenic differentiation of hBMSCs by modulating the Wnt/β-catenin signaling pathway. GA-GelMA hydrogels promoted bone fracture healing. GA has potential as a cost-effective treatment of bone defects.

## Introduction

Bone defects or fracture non-union caused by high-energy injury, infection and bone tumors are common clinical problems in orthopedic and trauma surgery (Dimitriou et al., 2011; Fazzalari 2011; Giannoudis et al., 2011). The incidence of bone non-union has been estimated to be 5–10% (Zura et al., 2016). Bone marrow-derived stromal cells (BMSCs) have self-renewal capabilities, which are able to differentiate into all kinds of cell types, including osteoblasts, chondrocytes, adipocytes and myocytes (Ankrum et al., 2014). It is vital to promote the recruitment of bone marrow stromal cells (BMSCs) in the microenvironment surrounding a fracture defect and to induce osteogenic differentiation of MSCs to facilitate bone healing (Park et al., 2015; Gibon et al., 2016; Coutu et al., 2017).

Many strategies are used to promote bone healing. Bone autografting is the gold standard treatment for non-union fractures. However, autografting has a number of limitations, including limited availability, donor site morbidity and severe complications (Dekker et al., 2017). BMP-2 and BMP-7 are considered essential in bone healing, and have been approved for clinical use (Fassbender et al., 2014; Begam et al., 2017). Nevertheless, the high cost of these agents has limited their clinical application. Another promising method is gene therapy involving the transfer of target genes that strongly promote osteogenesis into the host genome. However, gene therapy is also associated with a number of problems, including high cost and concerns over biological safety (Atasoy-Zeybek and Kose 2018; Shapiro et al., 2018). Therefore, it is important to find a cost-effective and safe strategy to remedy bone defects.

**FIGURE 1:**
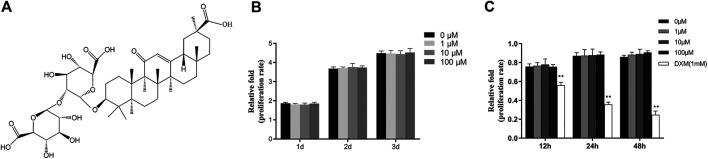
GA had no effect on the viability or proliferation of hBMSCs. **(A)** The chemical structure of GA. **(B)** The proliferation of hBMSCs was measured by CCK-8 assay after addition of different concentrations of GA (0–100 μM) for 1, two or 3 days in hBMSCs growth medium. **(C)** The proliferation of hBMSCs was measured by CCK-8 assay after addition of different concentrations of GA (0,1,10 and100 μM) and dexamethasone (DXM 1 mM) for 12, 24 and 48 h in hBMSCs basal medium with 1% FBS. The proliferation rate was normalized relative to that of group GA 0 μM.

Many small molecular compounds derived from natural products, which are abundant and inexpensive, with good pharmacological benefits and potential for clinical application. Liquorice is an extremely important Chinese herbal medicine that has been widely used for millennia due to its palatable taste and medicinal potential. Glycyrrhizic acid (GA) is a major triterpene glycoside isolated from liquorice root (Feng et al., 2015; Sun et al., 2019), and has been reported to have a variety of pharmacological activities, including anti-viral, anti-tumour, anti-inflammatory and anti-oxidative activities (Bhattacharjee et al., 2012; Su et al., 2017; Sun et al., 2019; Wang et al., 2011). GA, consisting of two molecules glucuronic acid and one molecule of glycyrrhetinic acid, has an amphiphilic structure and carboxyl and hydroxyl groups (Figure 1A). However, there has been little research regarding use of GA in bone regeneration. Yin et al. (Yin et al., 2019) reported that GA effectively inhibited osteoclast maturation and bone resorption and exhibited an osteoprotective effect in ovariectomy mice. Li et al. (Li et al., 2018) reported that GA inhibited RANKL-induced osteoclastogenesis. It suggested GA maybe beneficial to osteogenesis. The effects of GA in osteoclastogenesis have been explored. However, there have been no studies regarding the influence of GA on osteogenesis. Therefore, this study was performed to explore the effects of GA on the proliferation and osteogenic differentiation of human bone marrow stromal cells (hBMSCs) as well as the underlying mechanisms.

## Materials and Methods

### Reagents

GA (purity, > 97.5%, as determined by high-performance liquid chromatography [HPLC]) was purchased from TargetMol (Wellesley Hills, MA, United States). The GA was dissolved in dimethyl sulfoxide (DMSO) and stored at −20°C in the dark. Photopolymerisable gelatine methacryloyl (GelMA) hydrogels were purchased from Engineering For Life (Suzhou, China). XAV-939, an inhibitor of the Wnt/β-catenin signaling pathway, was purchased from Selleck Chemicals (Houston, TX, United States). We used 10 μM XAV-939 based on a previous study (Iwatani et al., 2017).

### Cell Isolation and Culture

hBMSCs were isolated from the whole bone marrow of two healthy donors (two men, aged 20 and 22 years) using the method described previously (Zhang et al., 2018). All donors provided informed consent before collection of their bone marrow and the protocol was approved by the Ethics Committee of the Second Affiliate Hospital of Zhejiang University. All of the treatment procedures conformed to the ethical standards of the Declaration of Helsinki. Adherent hBMSCs were then cultured in culture flasks with hBMSC growth medium (Cyagen Biosciences, Guangzhou, China) consisting of human bone marrow stromal cells basal medium, 10% human bone marrow stromal cell-qualified fetal bovine serum, 1% penicillin-streptomycin and 1% glutamine in an incubator at 37°C with 5% CO_2_, with passage after reaching 80% confluence. Cells from passages 3–5 were used in subsequent experiments.

### Cell Viability and Proliferation Assay

To assess the effects of GA on the proliferation and viability of hBMSCs, cells were seeded into 96-well plates (5,000/well) and allowed to adhere for 12 h. For the assessment of the proliferation of hBMSCs, the medium was then changed with hBMSCs growth medium, and the cells were subsequently treated with various concentrations of GA (0, 1, 10, 100 μM) for 1, 2 or 3 days. For the assessment of the viability of hBMSCs, the medium was then changed with hBMSCs basal medium with 1% FBS, and the cells were subsequently treated with various concentrations of GA (0, 1, 10, 100 μM) and dexamethasone (DXM 1 mM) for 12, 24 or 48 h.

The medium was then changed to 10% Cell Counting Kit-8 (CCK-8; Dojindo, Kumamoto, Japan) in 100 μL of low-sugar Dulbecco’s modified Eagle’s medium (L-DMEM) without fetal bovine serum (FBS) (Gibco, Waltham, MA, United States) for 4 h at 37°C. The absorbance at 450 nm (A_450_), which is proportional to the number of living cells, was measured using a microplate reader (ELX808; BioTek, Winooski, VT, United States).

### Osteogenic Differentiation Protocol

Osteogenesis of hBMSCs was induced by osteogenic induction medium (OIM) consisting of L-DMEM, 10% FBS, 100 IU/ml penicillin/streptomycin, 100 nM dexamethasone, 0.2 mM ascorbic acid and 10 mM β-glycerophosphate. Firstly, hBMSCs were cultured in hBMSCs growth medium in 6- or 12-well cell culture plates at a density of 3 × 10^4^/cm^2^ and incubated at 37°C in an atmosphere of 5% CO_2_. After cells reached about 80–90% confluence in the culture of hBMSCs growth medium, the culture medium was aspirated off and replaced with fresh OIM at different concentrations of GA ranging from 0 to 100 μM. The cells were maintained by the addition of fresh OIM every 2–days.

### ALP Staining and ALP Activity Assay

To investigate early mineralization, hBMSCs were treated with OIM and GA at different concentrations in 12-well plates for 3 days. For ALP staining, cells were fixed with 4% paraformaldehyde (Sangon Biotech, Shanghai, China) for 30 min. Cells were then washed with double distilled water (ddH_2_O) three times and stained using an Alkaline phosphatase Color Development Kit (Beyotime, Shanghai, China). ALP activity was determined by the ALP Activity Assay (Beyotime) according to the manufacturer’s instructions. Briefly, cells were lyzed about 1 h with RIPA buffer. The supernatant was collected used to further detection. The proper amount of supernatant and 50 μL chromogenic substrate (Para-nitrophenyl phosphate (pNPP)) were added in the wells on 96-well, which was complemented with testing buffer to 100 μL total system. And at the same time, we prepared the standard samples (para-nitrophenol 0.5 mM) that were used to prepare the standard curve of ALP. Then the 96-well plate was incubated at 37°C for 5–10 min. Finally, each well was added 100 μL reaction termination solution to stop the reaction and the absorbance 405 nm (A_405_) was measure by microplate reader.

### Alizarin Red Staining and Quantification Assay

Alizarin red staining (ARS; Cyagen Biosciences) was performed to assess late mineralization. hBMSCs were seeded in 12-well plates and cultured with OIM and GA at various concentrations. After 14 days of culture, cells were fixed in 4% paraformaldehyde for 20 min at room temperature and subsequently washed three times with ddH2O. Finally, the cells were treated with ARS (0.5%, pH 4.1–4.2) for 20 min and then rinsed with distilled water. To quantify the staining, stained mineralized nodules were incubated with 10% cetylpyridinium chloride (Sigma, Shanghai, China) Then the solution was collected and the A570 was measured microplate reader.

### Western Blotting Analysis

Cells were lyzed in RIPA buffer supplemented with proteasome and phosphatase inhibitors (Beyotime). Equal amounts of proteins were separated by 10% SDS-PAGE and then transferred onto polyvinylidene fluoride (PVDF) membranes (Millipore, Shanghai, China). After blocking in 5% non-fat milk for 1 h, the membranes were incubated overnight at 4°C with antibodies specific to GAPDH (1:1,500; CST#5174, Cell Signaling Technology, Danvers, MA, United States), COL1A1 (1:1,000; ab34710, Abcam, Cambridge, United Kingdom), RUNX2 (1:1,600; ab192256, Abcam), active *β*-catenin (1:1,000; ab246504, Abcam) or total *β*-catenin (1:1,000; ab223075, Abcam). A stripping method was used to measure the two antibodies of same molecular weight. After washing four times (5 min each time) in Tris-buffered saline with 0.1% Tween 20 (TBST), the membranes were incubated with horseradish peroxidase-conjugated secondary anti-mouse or anti-rabbit antibodies (Beyotime) for 1 h at room temperature. After washing three times (5 min each time) with TBST, proteins were detected using enhanced chemiluminescence blotting reagents (Millipore). Signal intensity was measured using a Bio-Rad XRS chemiluminescence detection system (Bio-Rad, Hercules, CA, United).

### Immunofluorescence Assay

Cells were cultured in 12-well plates with OIM. After induction of osteogenesis, cells were fixed in 4% paraformaldehyde for 15 min at room temperature, permeabilized in 0.05% Triton X-100 for 30 min and blocked in 2% bovine serum albumin (BSA) for 30 min. Fixed cells were washed and incubated overnight with anti-RUNX2 (1:500; Abcam) or anti-COL1A1 (1:500; Abcam) antibody. The cells were then incubated with fluorescence-conjugated secondary antibody (DyLight 550 Conjugate; Boster Biological Technology, Wuhan, China) for 2 h and nuclei were stained with 2-(4-amidinophenyl)-6-indolecarbamidine dihydrochloride (DAPI) (Beyotime) for 5 min. Samples were observed under a fluorescence microscope (EU5888; Leica, Wetzlar, Germany).

### RNA Isolation and Real Time Quantitative PCR

Total RNA was isolated from cells cultured with OIM using RNAiso reagent (Takara Bio Inc., Dalian, China) and quantified by measuring the A_260_ (NanoDrop 2000; Thermo Fisher Scientific, Waltham, MA, United States of America). First strand cDNA was synthesized using PrimeScript RT Master Mix (Takara Bio Inc.) according to the manufacturer’s instructions. Total RNA (≤1,000 ng) was reverse-transcribed into cDNA in a reaction volume of 20 μL using a Double-Strand cDNA Synthesis Kit (Takara Bio Inc.). The levels of mRNAs encoding COL1A1, RUNX2, OCN and GAPDH were determined using a StepOnePlus real-time PCR system (Applied Biosystems Inc., Warrington, United Kingdom) and SYBR Premix Ex Taq (Takara Bio Inc.) under the following conditions: 95°C for 30 s followed by 40 cycles of 95°C for 5 s and 60°C for 30 s. GAPDH was used as an internal control and allowed adjustment of differences among samples. DNA concentrations were calculated using the 2^−ΔΔCt^ method (Livak and Schmittgen 2001). All primers used in this experiment were synthesized by Sangon Biotech and are listed in Table 1.

**TABLE 1:** Sequences of primers for real-time quantitative PCR analysis.

Gene name	Forward primer (5‘→3‘)	Reverse primer (5‘→3‘)
COL1A1	CAG​ATC​ACG​TCA​TCG​CAC​AAC	GAG​GGC​CAA​GAC​GAA​GAC​ATC
RUNX2	TGG​TTA​CTG​TCA​TGG​CGG​GTA	TCT​CAG​ATC​GTT​GAA​CCT​TGC​TA
OCN	CAC​TCC​TCG​CCC​TAT​TGG​C	CCC​TCC​TGC​TTG​GAC​ACA​AAG
GAPDH	GGA​GCG​AGA​TCC​CTC​CAA​AAT	GGC​TGT​TGT​CAT​ACT​TCT​CAT​GG

### Fabrication of GelMA, GA/GelMA Scaffolds and Structural Characterization

Fabrication of GelMA was performed in accordance with the manufacturer’s instructions. Briefly, the GelMA powder was dissolved in 0.25% (w/v) lithium acylphosphinate salt (LAP) as a photoinitiator with or without GA. The hydrogel was then sterilized with a 0.22 μm polyethersulfone (PES) membrane syringe filter (Guangzhou Jet Bio-Filtration, Guangzhou, China). The concentration of GelMA was 10%, and the concentration of GA in the 10% GA/GelMA was 2 mM. Finally, the material was crosslinked using a 365-nm ultraviolet (UV) lamp with a light intensity of 2.7 mW/cm^2^ for 15 s and lyophilized for 24 h. The surface structure of samples was examined by scanning electron microscopy (SEM) (FEI Q45; FEI Co., Portland, OR, United States).

### Lentiviral Packaging and Cell Infection

A lentiviral green fluorescent protein (GFP) vector was purchased from Cyagen Biosciences and transfections were performed according to the manufacturer’s instructions. Briefly, hBMSCs were incubated with GFP lentiviral particles and 5 μg polybrene/mL in growth medium. After 18 h, the infection medium was replaced with hBMSC growth medium. After 2 days, the GFP activity were observed by fluorescence microscopy (EU5888; Leica Camera, Wetzlar, Germany).

### GFP-hBMSC 2D and 3D Culture in GelMA

For 2D culture, 10% GelMA was spread over 48-well plates and crosslinked using a 365-nm UV lamp. GFP-hBMSCs were then seeded on the surface of GelMA and cultured in growth medium at 37°C in an atmosphere of 5% CO_2_. For 3D culture, the cultured hBMSCs were trypsinized and collected by centrifugation. The resulting pellets were introduced onto 10% GelMA pre-solution and 0.25% (w/v) LAP as a photoinitiator, and sterilized with a 0.22 μm polyether sulfone (PES) membrane syringe filter (Guangzhou Jet Bio-Filtration). Finally, the material was crosslinked using a 365 nm UV lamp and washed twice before culturing the hydrogels in hBMSC growth medium at 37°C in an atmosphere of 5% CO_2_.

### *In vivo* Evaluation in Animals

All animal experiments were conducted in accordance with principles and procedures approved by the Institutional Animal Care Use Committee at the second affiliated hospital of Zhejiang University (approval number: 2020-No.25). A mouse femoral fracture model was created as described previously (Murata et al., 2014; Johnson et al., 2018) in a total of 15 8-week-old male wild-type C57BL/6 mice (SLAC Laboratory Animal, Shanghai, China). The animals were randomly divided into three groups: control group, GelMA hydrogel group and GA-GelMA hydrogel group. All surgical procedures were performed by two experienced orthopedic surgeons. Briefly, the mice were anaesthetised with 2% pentobarbital and the hair on the left hind leg was shaved. The skin was disinfected by swabbing with alcohol. A lateral incision was made over the femur and the muscle was blunt dissected to expose the femur. The patella was then dislocated and a sterile 25-gauge needle was inserted into the femur shaft and retracted. The needle was positioned through the femur to stabilize the fracture section at the middle and lower segment that was created using a custom-made three-point bender. Then, 3 μL of GelMA with and without GA was pipetted over the fracture site and polymerized with UV irradiation for 15 s. The control group received an equal volume of normal saline (NS). Finally, the muscle, patella and skin were sutured back into place. All the mice were killed one month after operation and the sample were used in the subsequent study.

### Radiographic Analysis

Animals were sacrificed at 1 month after surgery and the samples were collected and fixed in 4% paraformaldehyde for 72 h at room temperature. A range of 3 mm above and below the fracture tip was selected for scanning by micro-computed tomography (micro-CT). Femoral samples were scanned for bone formation using a μCT-100 Imaging System (Scanco Medical, Brüttisellen, Switzerland) with the following scan parameters: 70 kVp, reconstruction matrix of 1,024 and slice thickness of 14.8 μm with an exposure time of 300 ms. The trabecular bone volume fraction (BV/TV) were evaluated by 3D standard microstructural analysis (Bouxsein et al., 2010).

### Histological Evaluation

After micro-CT, all samples were decalcified using 10% ethylene diamine tetra acetic acid (EDTA; Sigma) in 0.1 M PBS, changing the solution once a week for 6 weeks, before embedding in paraffin. Serial sections 3 μm thick were cut and mounted on polylysine-coated slides, deparaffinized and then stained with hematoxylin and eosin (HE), Masson’s trichrome, Safranin O and fast green separately on consecutive tissue sections based on our previous studies (Chen et al., 2017). Images were obtained using a microscope (Leica DM4000B; Leica, Wetzlar, Germany). We used a scoring scale for histological quantification (Xue et al., 2017) based on cortical debridement and healing acceleration (Table 2). All evaluations were performed in a triple-blind manner.

**TABLE 2:** Histological evaluation scoring scale of fracture healing.

Scores	Histological evaluation sites
0	No bridging, no woven bone
1	No bridging, a small amount woven bone
2	No bridging, obvious initial woven bone near fracture
3	No bridging, marked woven bone near and around fracture site
4	Bridging of at least one of the cortices, marked woven bone near and around fracture site
5	Bridging of at least one of the cortices, marked and complete woven bone around fracture site
6	Bridging of both cortices, and/or some resolution of the woven bone
7	Clear bridging of both cortices and resolution of the woven bone

### Data and Statistical Analysis

All statistical analyses were performed using GraphPad Prism (version 8.0; GraphPad Software, San Diego, CA, United States). All experiments were conducted at least three times and the data are presented as means ± SD. Differences between two groups were analyzed by two-tailed Student’s *t* test. For comparisons between more than two groups, one-way analysis of variance (ANOVA) followed by the Bonferroni post hoc test was used. In all analyses, *p* < 0.05 was taken to indicate statistical significance.

## Results

### GA did not Aaffect the Viability and Proliferation of hBMSCs

We performed CCK-8 assay to assess the effects of GA on proliferation of hBMSCs. The hBMSCs were cultured in the hBMSCs growth medium with different concentration (0, 1, 10 and 100 μM) of GA for 1, 2 and 3 days. No significant effect of GA on the proliferation of hBMSCs was observed (Figure 1B). To assess the viability of GA on the hBMSCs, the hBMSCs were cultured in the hBMSCs basal medium with 1% FBS with different concentration (0, 1, 10 and 100 μM) of GA and dexamethasone (DXM 1 mM) for 12, 24 and 48 h. There had no significant differentiation of GA compared to the control group (0 μM). The group of DXM (1 mM) had obvious decrease of cell number (Figure 1C). It indicated that GA has no promoting or inhibiting effect on the proliferation of hBMSCs. Furthermore, GA has no unpromized effect on the viability of hBMSCs.

### GA Increased the Expression of Osteo-Specific Genes and Proteins and Enhanced Calcium Deposition

To assess the role of GA in osteogenic differentiation of hBMSCs, osteo-specific gene and protein expression were examined by real time quantitative PCR, western blotting and immunofluorescence analyses.

**FIGURE 2:**
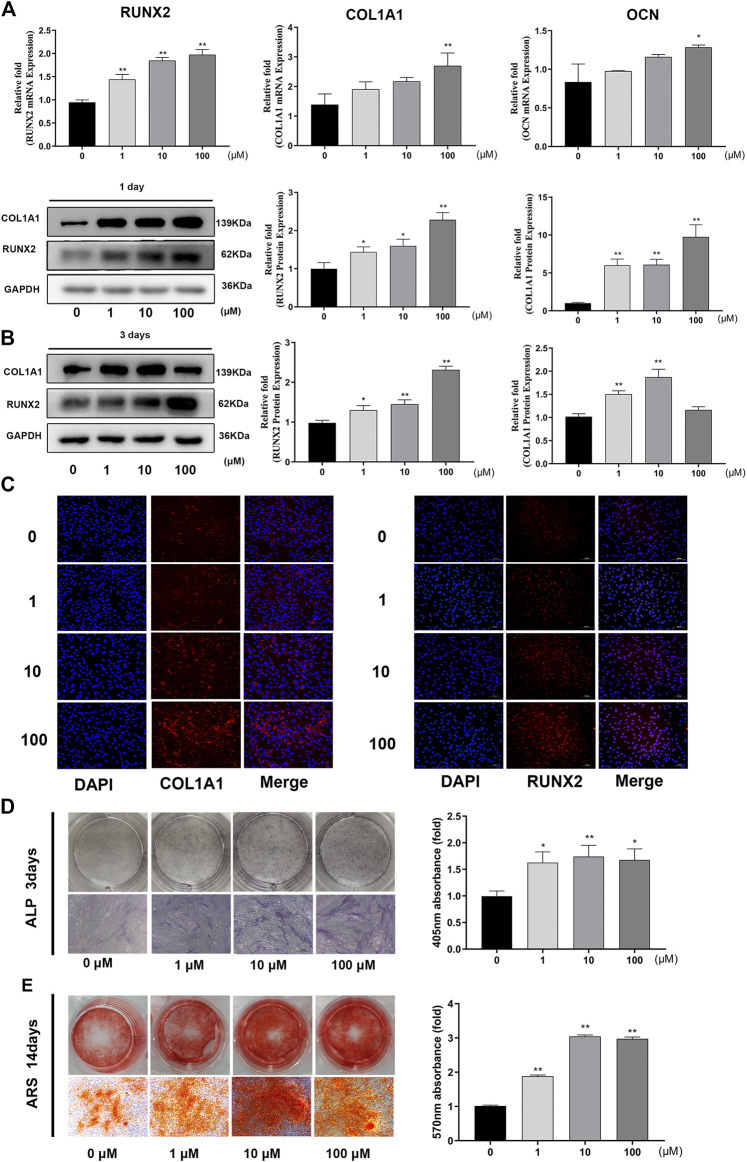
GA increased the expression levels of osteo-specific genes and proteins and enhanced calcium deposition. **(A)** Relative mRNA expression of osteo-related genes (RUNX2, COL1A1 and OCN) measured by real time quantitative PCR after 1 day of osteogenesis. The mRNA expression levels were normalized relative to that of GAPDH RNA. **(B)** Relative protein expression levels of osteo-related protein (RUNX2 and COL1A1) were measured by western blotting after 1 and 3 days of osteogenesis. The protein expression levels were normalized relative to that of GAPDH protein. **(C)** Immunofluorescence staining for COL1A1 and RUNX2 protein after 1 day of osteogenesis. Scale bars, 100 μm. **(D)** ALP staining and quantitative assay on day 3 of osteogenic differentiation. Scale bar, 500 μm. **(E)** Mineralization was measured by ARS staining and quantitative assay after 14 days of osteogenesis. Scar bar, 500 μm. All data are expressed as the means ± SD. Reactions were performed in triplicate. *p < 0.05, **p < 0.01 compared to the control group.

The expression levels of osteo-related genes (RUNX2, COL1A1, OCN) were markedly increased following GA treatment after 1 day of osteogenesis. The capacity of GA to promote osteogenesis varied at different concentrations. For RUNX2, COL1A1 and OCN, 100 μM was the maximal concentration to produce a significant difference (*p* < 0.05) (Figure 2A). Western blotting and immunofluorescence analyses indicated that the expression levels of osteo-related proteins (RUNX2 and COL1A1) were increased significantly compared to the control group on days 1 and 3 of osteogenesis. The effect of GA on the expression of osteo-related proteins (RUNX2 and COL1A1) was an increased dose-dependent relationship (Figures 2B,C).

We investigated the influence of GA on early stage mineralization during osteogenic differentiation by ALP. The results indicated that GA enhanced ALP activity on day 3 in a dose-dependent manner (Figure 2D) Late-stage calcium deposition and mineralized nodule formation was conducted by ARS. Similar to ALP, it showed that GA increased the calcium deposition and mineralization on day 14 (Figure 2E).

### GA Promotes Osteogenic Differentiation of hBMSCs Partly via the Wnt/β-Catenin Signaling Pathway

**FIGURE 3:**
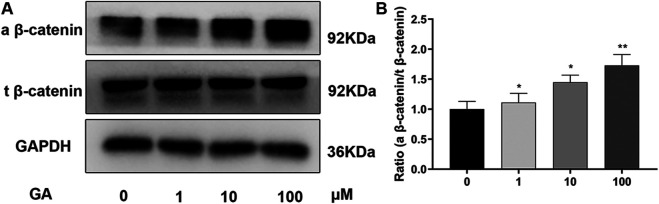
GA promotes osteogenic differentiation of hMSCs partly via the Wnt/β-catenin signaling pathway. **(A)** Relative protein expression levels of components of different signaling pathways were assessed by western blotting. hBMSCs were incubated with different concentrations of GA during osteogenic differentiation on day 3. The protein expression levels were normalized relative to that of GAPDH protein. **(B)** the quantitation of ratio (a beta-catenin/t beta-catenin) to reflect the activation of Wnt/beta-catenin signaling pathway. All tests were performed in triplicate. Data are expressed as means ± SD. **p < 0.01 compared to the control group.

To explore the pathway underlying the regulation of osteogenic differentiation of hBMSCs by GA, we examined the Wnt/β-catenin signaling pathway that is vital in osteogenesis. The expression of related proteins was investigated by western blotting (Figure 3A) on day 3 of osteogenesis. Increased expression of active β-catenin was observed in the GA treatment groups, which was significant at 10 and 100 μM (but not 1 μM), while there were no significant differences in expression of total β-catenin between groups. The ratio of active β-catenin and total β-catenin also significantly increased after treatment of GA. (Figure 3B). It indicated that Wnt/β-catenin signaling pathway maybe involved in the influence that GA promotes osteogenic differentiation of hBMSCs.

### Enhanced Osteogenic Differentiation of hBMSCs due to GA was Partially Attenuated by the Addition of Wnt/Catenin Signaling Inhibitor

**FIGURE 4:**
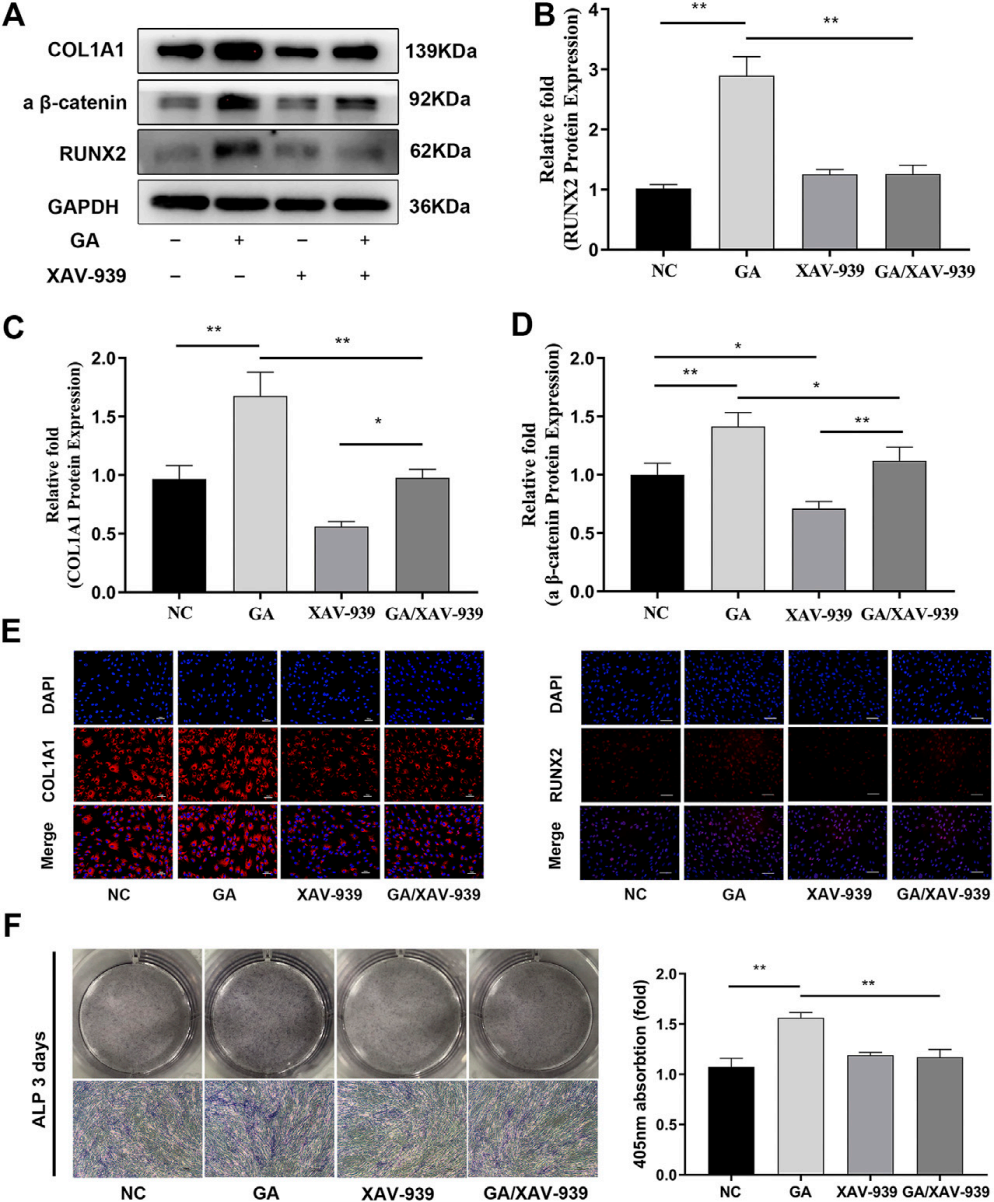
Enhanced osteogenic differentiation of hMSCs due to GA was partially attenuated by the addition of Wnt/catenin signaling inhibitor. **(A)** Inhibition of the Wnt/catenin signaling pathway partially attenuated the increases in RUNX2 and COL1A1 protein expression levels after addition of XAV-939 for 3 days. The protein expression levels were normalized relative to that of GAPDH protein. **(B–D)** Relative quantitative analysis of the western blotting results for active β-catenin, RUNX2 and COL1A1 protein. **(E)** Immunofluorescence staining after 3 days of osteogenesis. Scale bars, 100 μm. **(F)** ALP staining revealed decreased ALP activity in the GA/XAV-939 group compared to the GA group. Scale bar, 500 μm. Data are expressed as means ± SD. *p < 0.05 and **p < 0.01 between the two groups.

The Wnt/catenin signaling inhibitor XAV-939 was used to verify further the involvement of the Wnt/β-catenin pathway in the induction of osteogenic differentiation by GA. 10 nM of XAV-939 has been proved to inhibit the Wnt/catenin signaling pathway (Figure 4A). After the treatment of XAV-939, the increases in RUNX2 and COL1A1 expression induced by GA (100 μM) were attenuated as determined by western blotting (Figures 4A–D). Immunofluorescence analysis also showed that inhibition of the Wnt/β-catenin pathway partially attenuated the increases in expression of RUNX2 and COL1A1 by GA (Figure 4E). In addition, the enhanced mineralization due to GA was attenuated after addition of XAV-939 as determined by ALP assay (Figure 4E).

### hBMSCs Survived well in 2D and 3D Culture in GelMA Scaffolds that Showed Porous Structural Characteristics

**FIGURE 5:**
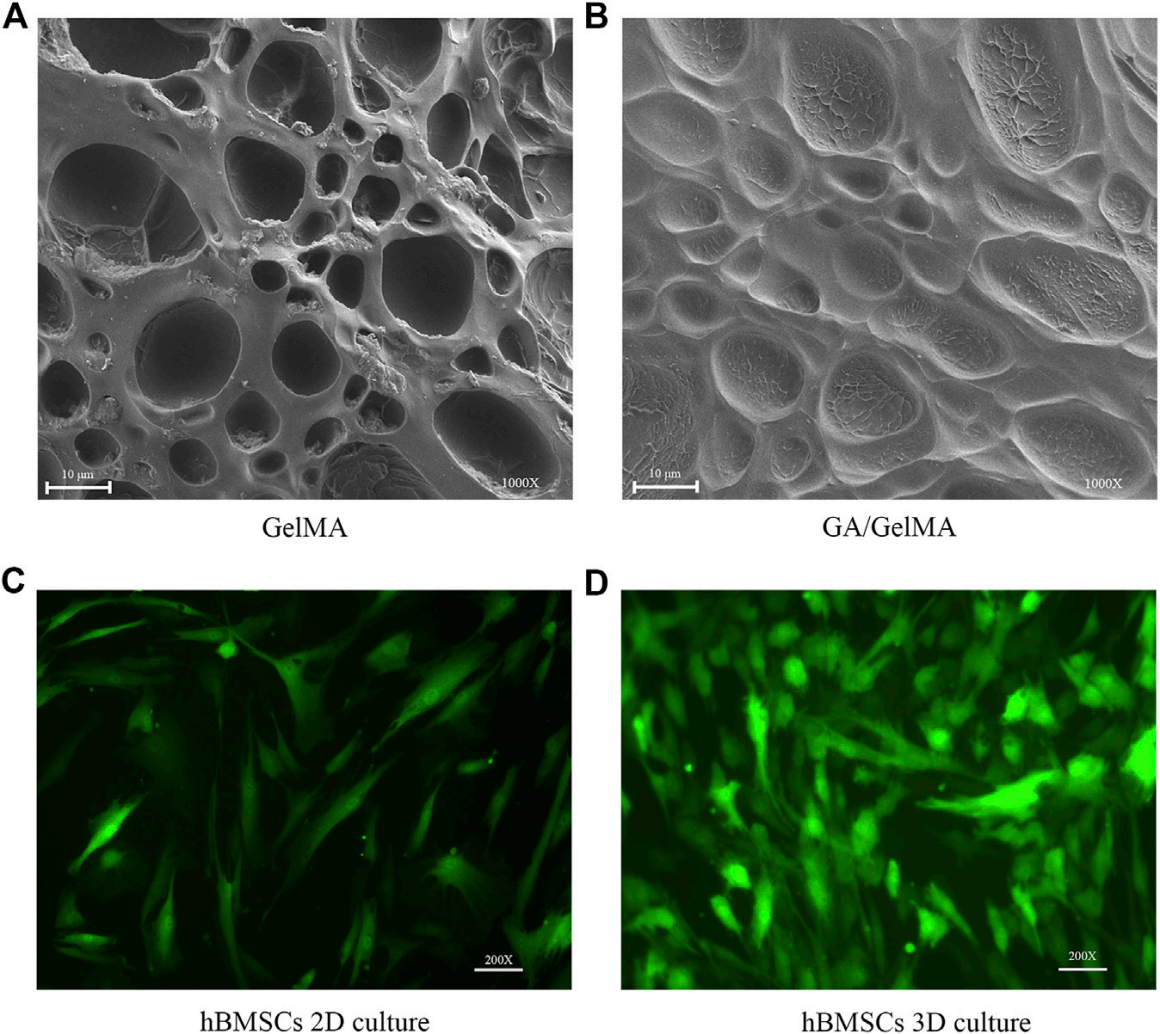
hBMSCs survived well in 2D and 3D culture in GelMA scaffolds that showed porous structural characteristics **(A,B)** The irregular elliptical pores structural features on the surfaces of GelMA and GA/GelMA. Scale bars, 10 μm. **(C,D)** Culture of GFP-hBMSCs at day 3 in 2D and 3D GelMA scaffolds. Scale bars, 100 μm.

We examined the structural characteristics of the surfaces of GelMA and GA/GelMA by SEM. GelMA and GA/GelMA both showed irregular elliptical pores that did not differ significantly in size between the two groups (10.17 ± 5.05 μm and 10.22 ± 5.57 μm, respectively, *p* > 0.5) (Figures 5A,B). It indicated the GelMA and GA/GelMA has well porous structural features, which provided a suitable surface structure for cell adherent growth. Furthermore, we investigated the survival of hBMSCs in GelMA. The results indicated that hBMSCs survived well and maintained their normal morphology in both 2D and 3D culture in GelMA (Figures 5C,D).

### GA-GelMA Hydrogels Accelerated Bone Healing in a Mouse Femoral Fracture Model

**FIGURE 6:**
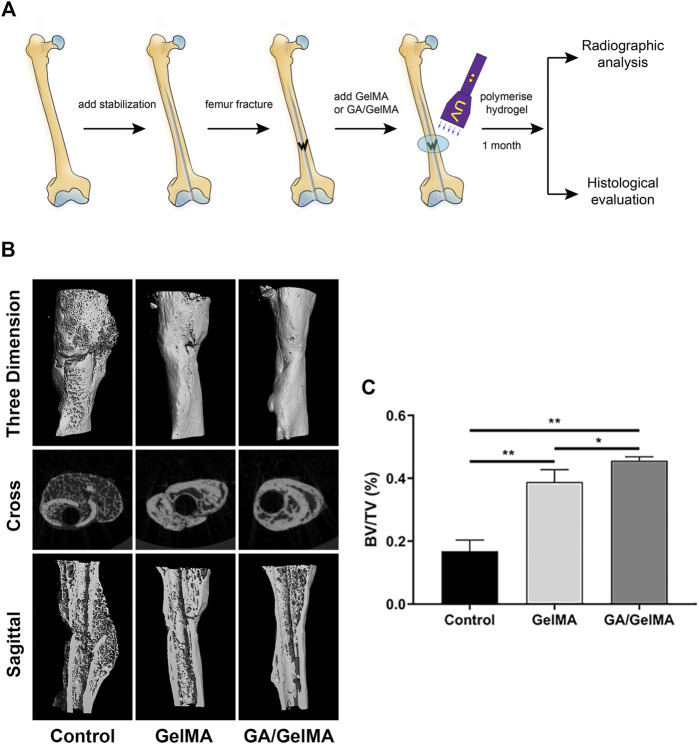
GA/GelMA hydrogels accelerated bone healing in a mouse femoral fracture model. **(A)** Schematic diagram of mouse femoral fracture model. **(B)** Micro-CT analysis of bone fracture healing. **(C)** Trabecular bone volume fraction (BV/TV) were analyzed by micro-CT. All experiments were performed in triplicate. Data are expressed as means ± SD. *p < 0.05 and **p < 0.01 between two groups.

**FIGURE 7:**
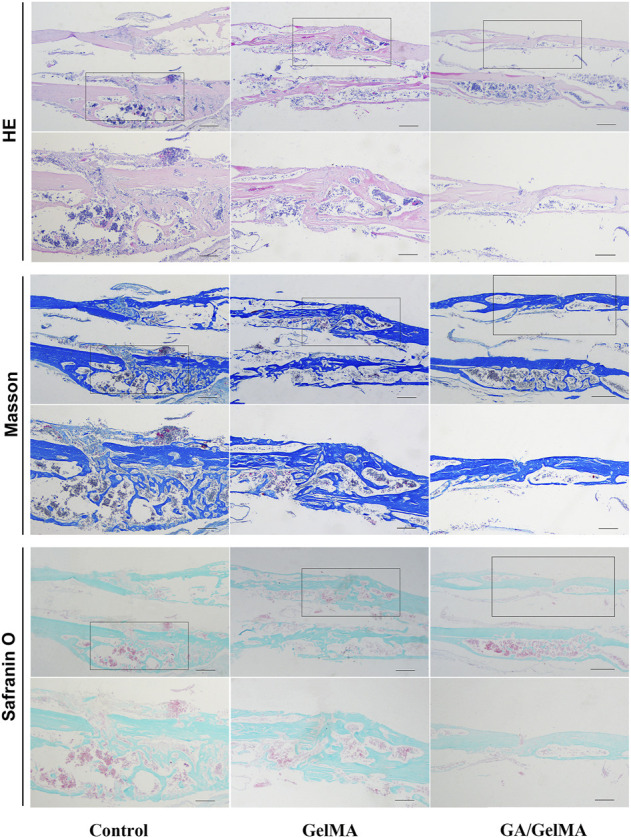
Histological evaluation, including HE, Masson’s trichrome, Safranin O and fast green staining of mouse femoral fracture area at 1 month after surgery. Scale bar, 500 μm.

**FIGURE 8:**
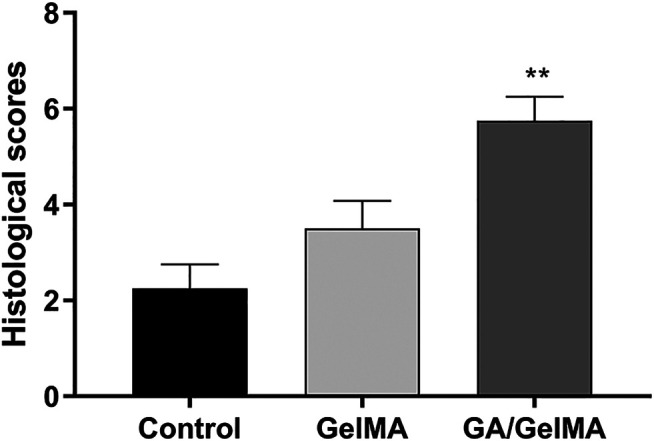
Histological evaluation score. GA/GelMA group had a significant increase in the histological evaluation score. Data are expressed as means ± SD. *p < 0.05 and **p < 0.01 between two groups.

To further assess the effects of GA *in vivo*, we used GA-GelMA hydrogels (GA/GelMA) in a mouse femoral fracture model. Micro-CT showed that GA/GelMA significantly accelerated bone fracture healing compared to control and GelMA groups (Figure 6B). One month after the operation, GA/GelMA significantly increased the BV/TV compared to the control group (Figure 6C). Furthermore, GelMA alone without GA also showed superior BV/TV values compared to the control group, although the GA/GelMA group showed significantly higher BV/TV values (Figure 6C). Histological evaluation, including HE, Masson’s trichrome, Safranin O and fast green staining revealed that the GA/GelMA group had better fracture union and cortical bridge compared to the control group and GelMA group (Figure 7). The GA/GelMA group showed significantly increased histological evaluation scores compared to the control group. (Figure 8).

## Discussion

To our knowledge, this is the first study to show that GA promotes osteogenic differentiation of hBMSCs partly by modulating the Wnt/β-catenin signaling pathway. We explored the influence of GA on the proliferation and osteogenic differentiation of hBMSCs. GA at concentrations from 1 to 100 μM neither promoted nor suppressed the proliferation of hBMSCs *in vitro*, which also had no effect on the viability on the hBMSCs, confirming its safety (Figures 1B,C). We found that RUNX2 and COL1A1 gene and protein expression levels were significantly increased after treatment with GA in a dose-dependent manner based on real time quantitative PCR, western blotting and immunofluorescence analyses (Figure 2). Furthermore, other osteo-specific genes, such as OCN also showed significantly increased expression with GA treatment. The effects of GA on early and late mineralization were also examined by ALP and ARS staining, which showed that GA enhanced the mineralization of hBMSCs in a dose-dependent manner. Therefore, GA significantly promoted osteogenic differentiation and enhanced the mineralization of hBMSCs. The *in vivo* study also indicated that GA can accelerate bone fracture healing in mice. It has been confirmed that GA can inhibit osteoclast formation and bone resorption function (Li et al., 2018; Yin et al. 2019). Bone homeostasis depends on the balance of osteoblasts and osteoclasts. Our study illustrated GA has dual beneficial role during the balance of bone homeostasis. Our study provides further information regarding the pharmacological actions of GA in the field of orthopedics. GA may have potential for the treatment of bone defects or non-union.

Most common signaling pathways involved in osteogenesis, including the Wnt/β-catenin, NF-κβ, PI3K-AKT and MAPK/ERK pathways. We detected the proteins of NF-κβ, AKT and ERK without significant differentiation (date not shown) which the activated Wnt/β-catenin pathway was observed (Figure 3). Wnt proteins are members of a family of secreted molecules that has been suggested to play roles throughout the healing process and in the promotion of osteoblast function (Krishnan et al., 2006). Wnt signals are transduced by a family of seven-transmembrane domain G protein-coupled receptors of the frizzled (FZD) family and a co-receptor of the arrow/LRP family (e.g., LRP5 and LRP6) or a Ryk or Ror transmembrane tyrosine kinase (Karner and Long 2017). Different Wnt proteins recognize their cognate receptors and activate at least three different intracellular signaling cascades: the canonical Wnt pathway (also known as the Wnt/β-catenin pathway), the noncanonical Wnt pathway and the Wnt-calcium pathway (Hang et al., 2019). The best characterized of these is the Wnt/β-catenin pathway. Wnt proteins activate the FZD/LRP5 or FZD/LRP6 receptor complexes and thus stabilize β-catenin in the cytoplasm Subsequently, β-catenin enters the nucleus and regulates the expression of target genes. GA has been reported the anti-viral, anti-tumours, anti-inflammatory and anti-oxidative. We think the strong anti-inflammatory of GA maybe has a relationship of the activation of Wnt/β-catenin signaling pathway. Therefore, we detected the Wnt/β-catenin signaling pathway. In our study, we detected higher ratio of expression of active β-catenin and total β-catenin after GA treatment during osteogenesis. Meanwhile, lower active β-catenin and osteogenic specific protein (RUNX2 and COL1A1) expression levels were observed after treatment with the Wnt/β-catenin inhibitor, XAV-939, compared to GA. We know PGE2 belong to the COX2 enzyme system mediates β-catenin transcription and COX2 inhibitor reduces beta-catenin cytoplasmic levels through ubiquitination and proteasomal destruction. GA maybe has a relationship of the activation of Wnt/β-catenin signaling pathway because of its’ activation of COX2 enzyme system. However, GA maybe just directly active the Wnt/β-catenin signaling pathway. Overall, these observations indicate that GA promoted the osteogenic differentiation of hBMSCs partly by modulating the Wnt/β-catenin signaling pathway.

Tissue engineering and regenerative medicine have emerged as promising strategies for bone reconstitution (Henkel et al., 2013). Various tissue-engineered scaffolds have been fabricated and applied in bone tissue engineering (BTE). Scaffold characteristics that can be modulated, improved or changed to make it more suitable for BTE applications must possess suitable biological and structural features, such as biocompatibility, no toxicity, biodegradability, bioactivity, osteo-inductivity and osteo-conductivity, as well as osteogenic features and mechanical properties (Roseti et al., 2017). Several different scaffolds have been developed, made of both natural and synthetic materials. Natural materials, such as collagen, silk fibroin and chitosan, show good biocompatibility and allow cell attachment and growth. However, natural materials have poor mechanical strength, making it difficult to control degradation time. Synthetic materials, including hydroxyapatite (HA), polylactic acid (PLA) and GelMA, have been used as scaffolds. However, HA does not have osteo-inductive properties and PLA has weak cell attachment capacity and poor mechanical strength. GelMA hydrogels have been used extensively for various biomedical applications requiring *in vivo* mimicry and 3D cell culture due to their suitable biological properties and tuneable physical characteristics (Yue et al., 2015). GelMA has RGD sites (Arg-Gly-Asp) for cell attachment, matrix metalloproteinase recognition sequences for biodegradability, excellent biocompatibility and tuneable physical properties (Choi et al., 2019). hBMSCs showed good survival in 2D and 3D culture in GelMA. Porosity and pore size are very important parameters in designing scaffolds for bone regeneration. SEM indicated that GelMA and GA/GelMA are porous, which contributes to osteogenesis. Ning et al. (Ning et al., 2019) reported that injectable photo-crosslinked GelMA hydrogel promoted the healing of bone defects in rats. However, pure GelMA hydrogel is difficult to greatly promote bone regeneration in the absence of any bioactive agent or pre-treatment (mineralization *in vitro*), limiting its application in bone repairing (Zheng et al., 2018). Therefore, GA/GelMA could increase BV/TV 6% relatively the pure GelMA. We designed a GA-delivered GelMA hydrogel, which was used in a mouse femoral fracture model, and the results of micro-CT and histological evaluation indicated that it had a superior effect on bone-fracture healing.

Several animal models have been used to study fracture non-union or delayed union (Chu et al., 2009; Lozada-Gallegos et al., 2013; Cappellari et al., 2014; Shimizu et al., 2015). In recent years, fracture research has focused on rats and mice (Garcia et al., 2013). However, the definitions of fracture non-union and delayed union are inconsistent (Garcia et al., 2013). Some researchers chose transverse osteotomy with additional periosteum resection to study fracture non-union or impaired fracture healing in mouse models. This approach may simulate open fractures with severe soft tissue injury, which are associated with a high incidence of non-union in clinical cases.

Our study had some limitations. First, GA modulated the Wnt/β-catenin signaling pathway to regulate osteogenic differentiation, and it is also likely involved in the activation of other signaling pathways. In addition to the canonical actions, non-canonical signals were not explored. Second, the underlying mechanisms, such as the cell-surface receptors that mediate signal transmission, were not clarified. Third, we investigated only endochondral ossification in our study, and further studies are required to determine the impact of GA on the processes of intramembranous ossification.

## Conclusion

In conclusion, our study showed that GA promotes the osteogenic differentiation of hMSCs by modulating the Wnt/β-catenin signaling pathway. GA-containing GelMA hydrogel effectively accelerated bone-fracture healing.

## Data Availability

The original contributions presented in the study are included in the article/Supplementary Material, further inquiries can be directed to the corresponding author.

## Abbreviations

GelMA hydrogels, Gelatin Meth-acryloyl hydrogels; ARS, Alizarin red staining; RANKL, receptor activator of nuclear factor κB ligand; BMP, bone morphogenetic protein; ALP, Alkaline phosphatase; GAPDH, glyceraldehyde-3-phosphate dehydrogenase; COL1A1, collagen type I a 1chain; RUNX2, runt-related transcription factor 2; OCN, osteocalcin; (active *β*-catenin, non-phosphorylated (active) *β*-catenin; t-β-catenin, total *β*-catenin; H&E, hematoxylin and eosin; BV/TV, trabecular bone volume per total volume; BTE, bone tissue engineering.
